# 

**Published:** 1849-11

**Authors:** 


☞ Notices of several new works are necessarily deferred.

